# Transforming *Tuta absoluta* Management: A Synergistic Approach Integrating Sustainability, Biological Control, and Biotechnological Innovations

**DOI:** 10.3390/insects16111173

**Published:** 2025-11-17

**Authors:** Abdul Basit, Farman Ullah, Muhammad Rehan Akhtar, Muhammad Humza, Muhammad Adeel Ghafar, Moazam Hyder, Inzamam Ul Haq, Youming Hou

**Affiliations:** 1 State Key Laboratory of Ecological Pest Control for Fujian and Taiwan Crops, Key Laboratory of Biopesticides and Chemical Biology, MOE, College of Plant Protection, Fujian Agriculture and Forestry University, Fuzhou 350002, China; malikbasituaf@gmail.com (A.B.); 2221903005@fafu.edu.cn (M.A.G.); 000b387972@fafu.edu.cn (M.H.); 000b370305@fafu.edu.cn (I.U.H.); 2 Xianghu Laboratory, Institute of Bio-Interaction, Hangzhou 311258, China; farmanullah787@gmail.com; 3 State Key Laboratory of Ecological Pest Control for Fujian and Taiwan Crops, Institute of Applied Ecology, Fujian Agriculture and Forestry University, Fuzhou 350002, China; m.rehan@fafu.edu.cn; 4 State Key Laboratory of Wheat Improvement, Shandong Provincial Key Laboratory of Agricultural Microbiology, College of Plant Protection, Shandong Agricultural University, Tai’an 271018, China; m_humza92@yahoo.com

**Keywords:** *T. absoluta*, microbial control, plant extract, RNAi, CRISPR/Cas9, SIT

## Abstract

*Tuta absoluta* (Meyrick, 1917), commonly known as the tomato pinworm, is a destructive pest of tomato and other solanaceous crops worldwide that leads to large yield losses. Because of the increasing resistance to chemical insecticides, alternative means of control are required. This review is focused on the actual integrated management with regard to natural, chemical, and advanced biotechnological control systems for *T. absoluta*. Traditional chemical methods of control, most commonly insecticides, have become less efficient as resistance develops. On the other hand, natural enemies such as predatory mirids, parasitoids, and microbial organisms seem to be promising in suppressing them. Moreover, biotechnology tools, such as RNA interference (RNAi), CRISPR/Cas9, and the Sterile Insect Technique (SIT), provide targeted and durable strategies against pests. Nano-bio-insecticides prepared using nanotechnological tools can enhance pest control through optimizing stability, toxicity, and release of active molecules. These improvements not only optimize pesticide monitoring but also contribute to sustainable farming. Integrated into a holistic IPM system, such innovative tools decrease dependence on chemical pesticides and offer environmentally friendly and effective solutions for *T. absoluta* control. This multi-modal approach outlines the need for further studies and the harnessing of these advanced techniques to suppress the emerging hazardous consequences posed by this invasive pest.

## Simple Summary

*Tuta absoluta* (Meyrick, 1917), commonly known as the tomato pinworm, is a key global pest of tomato, and high insecticide resistance is forcing the development of new control measures. This review presents current developments in integrated pest management strategies, which include the use of biocontrol agents and biotechnological strategies such as RNA interference (RNAi), CRISPR/Cas9, SIT, and nano-bio-insecticides. These approaches also present sustainable, environment-friendly solutions as an alternative to chemical insecticides, reducing the reliance on chemical controls and improving pest management in agro-food production.

## 1. Introduction

The South American tomato pinworm, *Tuta absoluta* (Meyrick, 1917) (Lepidoptera: Gelechiidae) is a notorious pest to South America [1] and it has become an important invasive pest that causes severe damage to tomato as well as other solanaceous crops. Aggregated damage in the majority of vegetables and broad-acre crops across more than 110 countries is attributed to *Tuta absoluta*, although it was first identified in eastern Spain back in 2006 [2,3,4,5,6,7]. This pest attacks many plants in the Solanaceae family, such as tomato, potato, eggplant, pepper, and tobacco. It is polyphagous, however, and readily feeds on several secondary hosts from the Amaranthaceae, Convolvulaceae, Fabaceae, and Malvaceae families [8]. Its wide range of hosts, high geographic spread, and adaptability under various environmental conditions make this pest a serious threat to agriculture worldwide and further highlight the importance of developing an effective IPM program.

*Tuta absoluta* is one of the most destructive insect pests of tomato, and it feeds on leaves, stems, and fruits, resulting in yield losses ranging from 80 to 100% if not managed. This pest also acts as a vector for the major inoculum source of tomato brown rugose fruit virus (ToBRFV) and, as such, it has substantial epidemiological importance in its field spread [9]. Its invasiveness is attributed to characteristics such as cryptic larval feeding, high fecundity, and multiple overlapping generations, coupled with strong dispersal capabilities. The pest status of *Tuta absoluta* is further complicated by its resistance to commonly used insecticides [10,11]. These biological and ecological characteristics render *Tuta absoluta* a major pest and thus require an appropriate integrated pest management (IPM) program for its control in tomato production.

As China is the largest tomato-producing country in the world, *Tuta absoluta* has spread rapidly over nearly 20 provinces of China since it was first found in Xinjiang in 2017 and then found in Yunnan in 2018 [12,13,14]. Given the abundance of good host plants—tomatoes, eggplants, and potatoes—and the presence of wood-boring hosts, it is quite probable that the pest will continue to expand its range. In 2021, the tomato production area of China exceeded 11 million hectares, accounting for approximately 22% of the global tomato industry [15]. Prediction models showed that *Tuta absoluta* might be able to migrate to most parts of China, except very cold regions such as Xizang, Qinghai, and Inner Mongolia [16,17]. Year-round survival is expected to be possible, even in a mild climate, due to the extensive use of protected locations for agricultural purposes [18]. The sudden rise in insect population densities represents significant risks for the economy and crop loss, high management costs, and trade hindrance [19]. Given the increasingly severe consequences mentioned above, *Tuta absoluta* was listed as a key quarantine pest and priority plant pest in 2023, which represents an important stage of the Chinese management strategy for agricultural pests.

Despite these sublethal effects, the early response to the invasion of *Tuta absoluta* has primarily been the application of chemical pesticides to mitigate *Tuta absoluta* infestation, which might affect various beneficial arthropods and other nontarget species [20]. Because they degrade producers, chemical insecticides, to sublethal and/or low lethal concentrations due to multiple biotic and abiotic factors, chemical insecticides have several sublethal effects on the targeted insects [21,22]. However, from several reported studies, nonchemical control tactics, including biological control, mass trapping, and planting of resistant cultivars, have shown a significant reduction in the use of chemical pesticides. Also, integrated application of control tactics is more efficient than using chemical insecticides, mainly in areas where *Tuta absoluta* is still early in the infestation stages [23,24,25,26]. Generally, because open-field crops are more reliant on chemical controls as compared to protected crops, it is hypothesized that protected crops are more targeted. Several national initiatives to develop and implement Integrated Pest Management strategies have been initiated in China since the first validated detection of *Tuta absoluta* in the country is assumed to be more sustainable mainly due to more diversified pest control.

## 2. Advancing Integrated Management Strategies for *Tuta absoluta*

### 2.1. Enhanced Strategies: Surveillance, Mass Trapping, and Mating Disruption

The densities of *Tuta absoluta* in tomato crops are often estimated through male trapping and/or egg and larval sampling. Although male density is often negatively correlated with tomato yield, the economic threshold based on male captures has hardly been defined due to the differences in trap efficiency, which is correlated with pest population density, trap type, the quality of the pheromone, and dispersal features [27]. In addition, adult captures can be unreliable, as their combination with egg counts on every crop was shown to be an uncertain method for monitoring damage loss [28]. Manual egg count has proven to be laborious because the eggs are small and uniformly distant, so a leaf-wise sampling is required in significant numbers. A non-cryptic binomial sampling method that depends on searching the middle third of the plant was determined to limit fruit damage below 1% [29].

The artificial female sex pheromone is the most widely used method for monitoring and mass trapping of *Tuta absoluta*. In an Argentine greenhouse tomato system, where the number of traps is 48 per ha, induced a significant reduction in leaf infestation compared to conventional insecticide treatments [30]. Pheromone dispensers and light and water traps have shown attraction of male and female adults, but glue boards are ideal for monitoring infestations since a significant proportion of non-targets are inadvertently caught [31]. Mating disruption with 30–60 g per hectare of pheromone led to high containment: near eradication conditions in greenhouses. The efficacy of this approach is heavily reliant on the closure of polyethylene greenhouses and drops considerably in open plastic houses, especially in the Mediterranean, since armand populations are significantly enhanced [32,33]. The only successful control measure is the physical exclusion of fecund females from greenhouses. Furthermore, SIT at a ratio of 15:1 has produced suitable results [34].

### 2.2. Tomato Resistance and Breeding Strategies

The breeding of tomato plants resistant to *Tuta absoluta* has also been a cornerstone of pest control since the 1990s due to this pest’s ability to cause about 90% crop loss in cultivated tomatoes. Genetic resistance has been discovered in some *Solanum lycopersicum* germplasm accessions [35,36], but the most effective sources of resistance so far are wild tomato species [37,38,39,40]. Breeding has concentrated on enhancing resistance mechanisms, which include increasing the level of leaf allelochemicals and enhancing trichome density [41,42]. Glandular trichomes that synthesize repellent chemicals, among which 2-tridecanone, zingiberene, and acyl sugars are the most important and are the most effective against *Tuta absoluta* [43,44,45,46]. These chemicals have both antixenosis properties, as they interfere with oviposition and larval feeding, and result in antibiosis effects that kill larvae [47,48,49]. While initial studies were focused on yeast-like 2-tridecanone elevation level, later investigations aimed to boost the level of zingiberene [50] and acyl sugars [42,51,52]. Lines with a higher concentration of acyl sugars were created, and the release of resistant cultivars is anticipated in the upcoming years [53,54].

Induced resistance has long been proposed and successfully used as one of the strategies to control *Tuta absoluta* on tomato. *Tuta absoluta* is a chewing insect and activates the jasmonic acid pathway, which often confers cross-talk between various defense signaling pathways, generally resulting in broad-spectrum resistance to various types of pests [43,55]. This mechanism may involve (a) the release of constitutive allelochemicals that are toxic to the herbivores, (b) the release of volatile organic compounds VOCs that attract natural enemies, and *Tuta absoluta* (c) suppression of the release of VOCs, so that the insect is unable to locate its host plant [56,57,58]. These findings suggest that resistance induced as a sustainable approach could be efficiently applied in IPM strategies.

**Figure 1:**
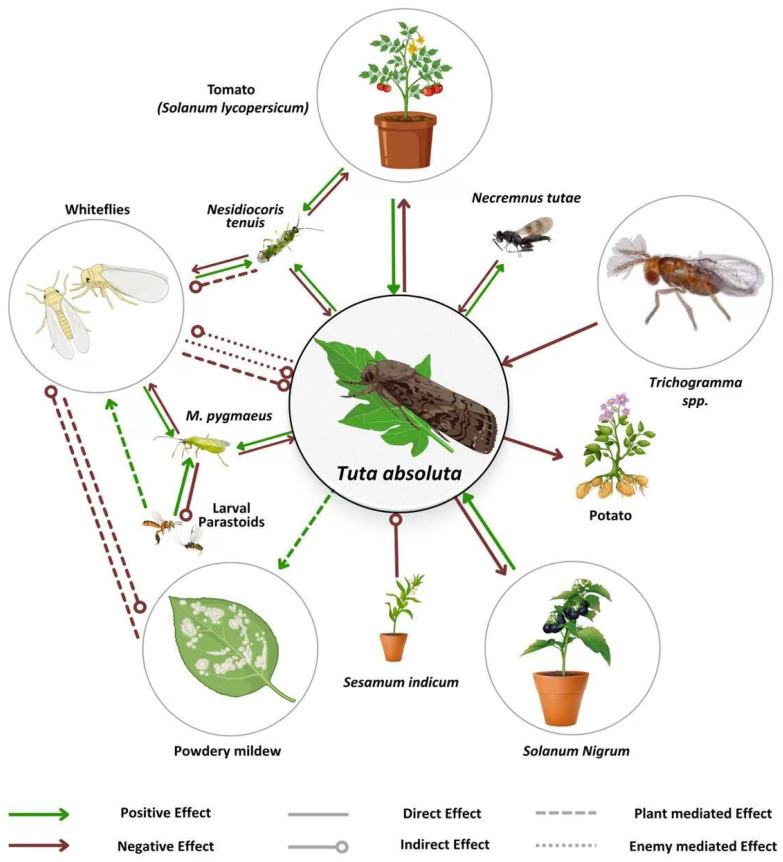
Depicts the biotic interactions between *Tuta absoluta* and various components of the tomato agro-ecosystem and neighboring systems. Negative effects are shown with lines and circles, while positive effects are represented by lines with arrows. Solid lines indicate direct interactions, and dashed lines represent indirect interactions, with green and red lines illustrating plant- and natural enemy-mediated effects, respectively. The thickness of the lines and the size of circles/arrowheads reflect the strength of these interactions. The signs and magnitudes of the interactions are derived from the studies cited in the review. Additional species involved in parasitoid interactions, such as *Stenomesius japonicus* and *Bracon nigricans*, are also included.

Such a calculation offers promise as a new avenue to manipulate tomato resistance to *Tuta absoluta*; however, it also comes with unknown consequences. Reliance on constitutive resistance alone might downplay some of the antixenotic traits associated with *Tuta absoluta* attacks and mutually exclusive to other pests. Plant resistance pathways are not isolated, and resistance expression plays out as a daily and spatial process affecting not only *Tuta absoluta* but all other pests and natural enemies that will share the same host species [59,60]. In fact, whiteflies induce plant-mediated negative interactions while slugs share the jasmonic and salicylic signaling and cross-talk defense pathways with *Tuta absoluta* [61,62], as shown in Figure 1. Consequently, the feasibility of manipulating resistance against *Tuta absoluta* will expand to alter the entire microorganisms and the plant-microbe community with unforeseen consequences and a bottom-up impact on yield.

## 3. Host Plant Resistance for the Control of *T. absoluta*

The management of *Tuta absoluta* has been on the priority list of international collaboration since the early 1990s, and breeding resistant lines to the pest in tomato plays a significant role in sustainable pest control [42]. *Tuta absoluta-resistant* wild tomato species are a valuable source of resistance genes for breeding resistant cultivars [53]. Glandular trichome-containing tomato lines are resistant against *Tuta absoluta*, whereas non-glandularly trichomed lines are highly susceptible [54]. This shows the relevance of morphological characters like trichome density for pest resistance. An increase in glandular trichome notch appealeness, even within genotypes with low overall trichome, can improve resistance [55]. Glandular trichomes release secondary metabolites such as jasmonic acid, which functions not only in insect repellence but also supports the production of natural predators of *Tuta absoluta* [54]. Additionally, glandular trichomes can cause mechanical obstruction to the larvae’s feeding and produce exudates that induce larval mortality. Thus, breeding tomato lines with higher levels of glandular trichomes can be a strategy for decreasing chemical pesticide use and developing potential tools for sustainable pest management.

The biochemical composition of tomato leaves is important for resistance to *Tuta absoluta* and differs between genotypes. Fruits with a large amount of flavonoids, phenolic contents, and tomatine are generally less infested, thus, these characteristics become handy in breeding for resistant varieties. Allelochemicals, e.g., acyl sugars, zingiberene, and 2-tridecanone, also aid in decreasing the number of eggs laid, decreasing the amount of plant damage, and reducing the percentage of probed age leaflets [39,43,63]. D’Esposito et al. reported a noteworthy negative relationship of *Tuta absoluta* infestation with phenolic contents in tomato leaves. Additionally, increased levels of anti-nutritional factors (reducing sugars) seemed to be involved in pest deterrence and were associated with higher infestation. Furthermore, tomato fruits with higher lycopene content were positively correlated with *Tuta absoluta* infestations, reflecting that the metabolite could act as an attractant for the insect to the fruit [60].

Studies conducted on various tomato cultivars showed that some of them, including Rio Grand VF, Matin, Pusa Ruby, and others, were resistant to *Tuta absoluta* [36,59,60,61,62]. According to Yang et al. (2024) [64] the modern variety Dafeng influences immature *Tuta absoluta* survival and performance, showing a faster preadult development but higher fecundity and a higher rate of intrinsic increase than those observed on processed tomato varieties. In contrast, Th9 is the most sensitive among processed cultivars, where other cultivars such as *Th1902*, *Heinz1015*, and *Dimen2272* have a lower sensitivity. These results are of importance for the commercial production with regard to choosing tomato cultivars that result in low infestations of *Tuta absoluta*, and facilitate an integrated pest management strategy.

The effect of the density of glandular trichomes on predation of *Tuta absoluta* by mirid predators, however, is inconsistent. Bueno et al. (2019) [65] showed that the effect of trichome density on predation was predator-specific, with *M. basicornis* showing a significantly greater impact compared to *E. varians* and *C. infumatus*, respectively. This means that pursuing high trichome density as a stand-alone resistance strategy may be a double-edged sword, since natural enemies’ potential to maintain low pest numbers can be undermined. Similarly, Bottega et al. (2017) [66] revealed that increased pubescence in the resistant tomato cultivars was inversely related to the predation of *P. nigrispinus*, a natural enemy of *Tuta absoluta*. The higher number of glandular trichomes, both type I and IV, in resistant genotypes compared with susceptible ones led to decreased predator survivorship, adult longevity, and larval consumption.

## 4. Cutting-Edge Strategies in the Biological Control of *T. absoluta*

Approximately 160 species were documented as natural enemies of *Tuta absoluta* in South America [67] and Afro-Eurasia [68], and 94.7% of those species were polyphagous. As such, developing a systematic approach of biological control measures against this pest had been difficult due to the absence of native natural enemies [69]. Nonetheless, when the pest successfully invaded Europe, conservation and augmentative biological control measures pursued in this regard brought the desired outcome. Significant efficacy in *Tuta absoluta* control has been recorded in both native and invaded lands by Hemiptera predators: anthocorids, geocorids, mirids, nabids, and pentatomids. In Europe, the sustainable management of the pest was rapidly established after its introduction by employing omnivorous mirids, in augmentative and inoculative releases within agricultural fields and plant nurseries [70,71,72], and efficient conservation approaches utilizing banker plants [73,74]. *Nesidiocoris tenuis*, *Macrolophus pygmaeus*, and *Dicyphus* spp. feed on *Tuta absoluta* eggs and larvae. This leads to almost complete abolishment of the prey reproduction in tomato crops [75].

Mirid predators have distinct habitat preferences and ecological roles. While *N. tenuis* has been more successful within the *Tuta absoluta*-tomato system [73], the generalist *Macrolophus pygmaeus* requires alternative prey, such as whiteflies, to thrive and complete development [74]. *Dicyphus* spp. also feed on *Tuta absoluta* eggs; however, their population growth rates are not as high when prey is less available [76,77]. Predators can also be harmful or neutral to plant health. Although *N. tenuis* can reach pest densities and cause necrologistic wall damage to plants and fruit in the absence of prey [71,78], *M. pygmaeus* and *Dicyphus* spp. have not been reported to cause similar plant damage [79,80]. The polyphagous nature of these mirids can also lead to interspecies interactions between the pest populations. For example, *M. pygmaeus-mediated* apparent competition between whiteflies and *Tuta absoluta* has been reported, and the introduction of *Tuta absoluta* in Europe has made biocontrol agents target native whitefly species [81].

Apart from predators other than mirids, such as *vespids* in Brazil [67,82], these could also be promising candidates for biocontrol of *Tuta absoluta*, though they have never been substantially incorporated into the IPM of the pest. Other taxa that have been occasionally observed to feed upon *Tuta absoluta* are spiders, predatory mites *Thripidae*, lacewings *Chrysopidae*, earwigs *Hemerobiidae*, ground beetles, ladybirds, and ants, but have not been studied deeply for their potential control role [68].

Several studies have considered the possibility of controlling *Tuta absoluta* with the help of various microbes. In particular, commercial strains of *Bacillus thuringiensis var. kurstaki* and *aizawai* showed larvicidal activity after ingestion [83]. Other microbial agents, such as different *Bacillus* spp. or fungi such as *Beauveria bassiana* and *Metarhizium anisopliae*, have been considered as well [84,85]. However, to date, no commercial biopesticides specific to *Tuta absoluta* have been created and registered. Nematodes of the genera *Steinernema* and *Heterorhabditis* have achieved the most significant results in suppressing the plant infestations by 87–95% under controlled laboratory and greenhouse conditions [86]. Nevertheless, the commercial introduction of these nematodes has not yet been achieved in greenhouses and fields.

**Figure 2:**
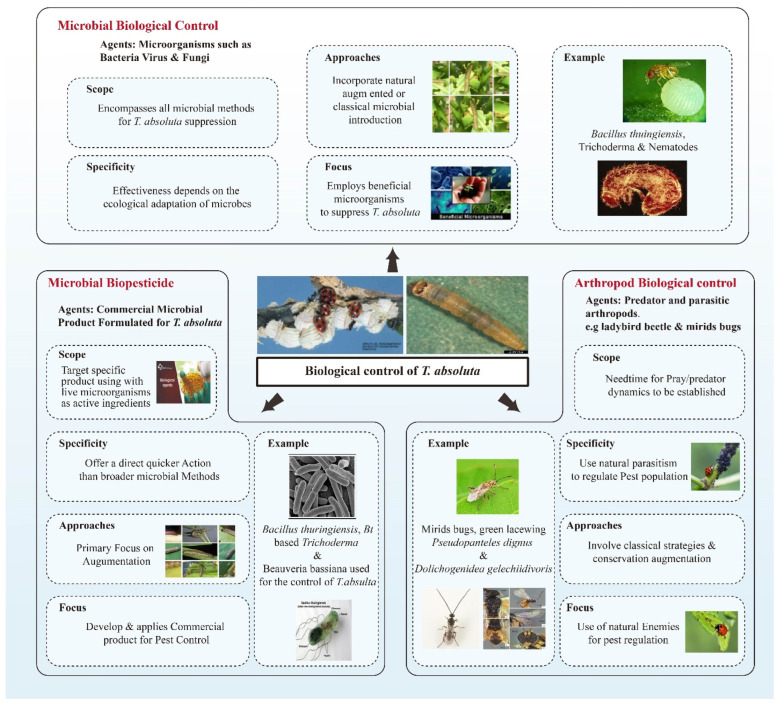
Various biological control methods for *Tuta absoluta*, a devastating pest of tomato plants. The diagram is divided into three main categories: microbial biological control, microbial biopesticides, and arthropod biological control. It explains the agents, scope, specificity, approaches, and focus for each method. The top section on microbial biological control highlights the use of microorganisms like bacteria and fungi for suppression, with examples including Bacillus thuringiensis, Trichoderma, and nematodes. The left section on microbial biopesticides focuses on commercial products formulated with live microorganisms, offering a faster action than broader methods. Examples provided are Bacillus thuringiensis, Beauveria bassiana, and Bi-based Trichoderma. The right section on arthropod biological control details the use of predator and parasitic arthropods, such as ladybug beetles and mirid bugs, to regulate pest populations through natural predation and parasitism. Visuals illustrate key examples and processes, creating a comprehensive overview of integrated pest management strategies for *Tuta absoluta* (Mawcha et al., 2025) [92].

For the optimal biological control of the *Tuta absoluta*, an integrated management program combining compatible biocontrol Agents, i.e., Parasitoids, microbial agents, e.g., Bt, and mirid predators, is advised [75,87]. Caution should be taken when involving natural enemies, as their presence may disrupt the whole biocontrol process [88]. The success of integrated releases is predicated on *IGP* reduction and the natural enemies’ compatibility and efficacy during the cropping season [88,89,90]. For instance, it has been observed that the parasitoid Trichogramma and the omnivorous predator *Macrolophus pygmaeus* possess dissimilar functional traits. Despite the predator’s kleptoparasitism effect, these natural enemies proved effective in controlling the pest when used together [91], as shown in Figure 2. Therefore, this approach seems to be the most attractive for explaining the actions to control pests.

## 5. Microbial Control and Biopesticides

Several microbial biological control agents, either bacteria, fungi, viruses, and entomopathogenic nematodes, have emerged as promising alternatives to manage *Tuta absoluta*. Either they infect and kill the pest or, under some conditions, compete with it for resources; they are considered a safe and sustainable option for pest control [7]. Despite proven effectiveness against *Tuta absoluta* in many studies, the number of works dedicated specifically to the evaluation of the effectiveness of the tested entomopathogens against the eggs and larvae of this pest is limited [3]. Numerous entomopathogens also show great potential in the laboratory, but only a few are used to prepare commercial biopesticides [91].

The most common and reliable bacterial biocontrol agents are *Bacillus thuringiensis* Bt strains. Several *Bt* strains have demonstrated excellent activity against *Tuta absoluta* at all larval instars with high levels of insecticidal activity [3,93,94]. In particular, the native *Bt* strains from Ethiopia, isolated from the local soil containing native crystalline proteins of the *Bt* strains, have demonstrated high efficiency, killing *Tuta absoluta* larvae up to 75% [95].

Fungal biocontrol agents, particularly *Metarhizium anisopliae* and *Beauveria bassiana*, have shown considerable potential in combating *Tuta absoluta*. Several *M. anisopliae* strains have been linked to high mortality in *Tuta absoluta*, including 37.14% mortality of females [91]. Some species of *B. bassiana* have demonstrated high mortality levels, with more than 90% of *Tuta absoluta* larvae succumbing to the fungus [96,97,98,99,100]. Additionally, 87%, 57%, and 66% of second-instar radish leaf miner larvae died when exposed to *M. anisopliae*, *Verticillium lecanii*, and *B. bassiana*, [101], respectively. Other fungal species within the genera *Isaria* [102], *Lecanicillium* [103], *Purpureocillium* [104], *Aspergillus* [105], and *Clonostachys* spp. [106] have also been used to control *Tuta absoluta*. *I. fumosorosea* is of particular interest because it led to a significant reduction in *Tuta absoluta* fecundity and longevity, limiting egg and larval output. In addition, a soil-related *A. oryzae* strain from Tanzania killed 77% of laboratory radish leaf larvae [105].

Granuloviruses—most notably *Phthorimaea operculella* granulovirus (PhopGV)—constitute potent, per os–active viral entomopathogens for *Tuta absoluta*, wherein ingested occlusion bodies dissolve in the larval midgut to liberate virions that establish a disseminated, ultimately lethal infection [107]. Complementarily, entomopathogenic nematodes (EPNs) exhibit pronounced activity within foliar tissues and galleries: *Steinernema carpocapsae*, *S. feltiae*, and *Heterorhabditis bacteriophora* suppress larval cohorts on leaves and in mines [86], with reported terminal mortalities of 97.4% for *S. feltiae* and 99% for *H. bacteriophora* against fourth instars [108,109]. Additional taxa—*H. baujardi*, *H. noenieputensis*, *S. jeffreyense*, and *S. yirgalemense*—expand the candidate palette for larval suppression, and *S. yirgalemense* uniquely achieves statistically robust pupal infection and kill [110]. EPN efficacy is strongly microclimate-dependent: *S. carpocapsae* manifests heightened virulence under higher thermal regimes (30–35 °C) and reduced humidity, whereas *S. feltiae* performs optimally at cooler conditions (15–20 °C) [109]. Collectively, these agent-specific thermal–hygrometric optima argue for climate- and phenology-aligned deployment and formulation to maximize field-level suppression.

The gut microbiota of *Tuta absoluta* potentially facilitates adaptive plasticity across heterogeneous environments. Remarkably, *Tuta absoluta* populations from Togo and Burkina Faso have remarkably conserved community profiles, with Proteobacteria and Firmicutes present in the highest abundances despite being geographically isolated [111]. Convergent microbiome composition likely harbors a functionally robust core microbiome that allows it to exhibit dietary flexibility, detoxification ability, and immune homeostasis, and it may have enabled its spread across heterogeneous agro-ecosystems. As a result, relationship-intensive interventions and microbiota-disruptive strategies represent promising directions for the development of future pest control.

Microbial biopesticides—bacterial, fungal, viral, and entomopathogenic nematode agents—thus represent compelling candidates for *Tuta absoluta* suppression. Compared to synthetic insecticides, bioinsecticides harbor less human toxicological risk and environmental persistence and form fewer residues in the trophic chain, yet offer orthogonal modes of action amenable to IPM deployment and resistance rotation. Acting via infection, toxicosis, or symbiont-mediated interference, biopesticides disorient pest physiology, behavior, and reproduction, generating multi-modal pressure on target populations to bolster the durability of control outcomes [112].

For the successful management of *Tuta absoluta,* use *Bacillus thuringiensis Bt*, *Metarhizium anisopliae*, *Beauveria bassiana*, and *Phthorimaea operculella granulovirus PhopGV* products constitute major microbial biopesticides, all with significant activity [7,107]. The harvests from them are globally marketed, especially *Bt*-based ones such as *Bt var. kurstaki Berliner* and *Bt var. aizawai*, both harmful to *Tuta absoluta* larvae. The formulations are applied as dusting powders, sprays, or granules and have consistently reduced foliar miner and feeder injury on tomato plants [113,114]. The product’s mode of action is that during sporulation, *Bt* produces Cry δ-endotoxins that are absorbed when ingested and that impair epithelial gut cell structure; the *Tuta absoluta* first instar is most sensitive [83]. The Salvadoran is an actinobacterium that produces spinosad and can be sprayed or dusted against *Tuta absoluta* spinosad, an additional microbial tool for integrated pest control [115].

Fungal biopesticides like *Metarhizium anisopliae* and *Beauveria bassiana*, on the other hand, are gaining more scientific ground for their capability to kill *Tuta absoluta* at all life stages, including eggs, larvae, and pupae [92]. However, these fungi use the host cuticle as an entry point into the body and then grow inside the host, causing the host to die [116]. Originating from prior research on other insect species, these biopesticides represent a breakthrough in the suppression of *Tuta absoluta*. For instance, *Tuta absoluta* populations have been successfully reduced by using the bred strain R444 of *B. bassiana* [92]. In addition to these fungal strains, other bioagents such as *M. anisopliae* and endophytic *Hypocrea lixii*, *Trichoderma* spp. show great potential and are already in the pipeline for the development of a targeted bioagent against *Tuta absoluta* [91]. In line with this, *Isaria fumosorosea* causes a significant reduction in *Tuta absoluta* female fecundity and adult longevity [117], and a soil-associated strain of *A. oryzae* from Tanzania attained up to 77 percent larval mortality under laboratory conditions [105]. These findings highlight the potential of bioagents in the fungi phylum as a decisively efficient approach to the management of *Tuta absoluta.*

The majority of *Tuta absoluta* larvae can be controlled by EPNs, such as *Steinernema carpocapsae*, *S. feltiae*, and *Heterorhabditis bacteriophora*, on tomato leaves or tunnel-grown populations [86]. For *S. feltiae* and *H. bacteriophora*, mortality was 97.4 and 99%, respectively, with fourth-instar larvae [108]. Other EPN species, including *H. baujardi*, *H. noenieputensis*, *S. jeffreyense*, and *S. yirgalemense*, were also very effective in managing *Tuta absoluta* larvae [110]. *S. carpocapsae* has increased virulence at high temperatures and low humidity, while *S. feltiae* is more effective at moderate temperatures. Thus, it is preferable to utilize these EPN species under field conditions when selecting EPN species based on the environmental situation in the target area.

*Tuta absoluta* gut microbiota has a critical effect on its capacity to colonize and disperse in multiple habitats. Although *Tuta absoluta* gut microbiome arises from different areas of the world, it has the common *phyla Proteobacteria* and *Firmicutes* since they are the most prevalent bacterial phyla [111]. This may imply that the gut microbial community functions enable the pest’s ecological success and spread, and may be a vital target for a new type of pest management.

## 6. Arthropod-Based Biological Control of *Tuta absoluta*

*Tuta absoluta* has been effectively controlled using a variety of arthropod natural enemies, both predators and parasitoids [68]. Approximately 60 generalist predators have been recorded, with the most important biological control agents being the mirid bugs *Nesidiocoris tenuis* Reuter and *Macrolophus pygmaeus* Rambur Hemiptera: *Miridae. Nesidiocoris tenuis* and *Macrolophus pygmaeus* are commercial species that are highly effective predators of *Tuta absoluta* eggs and early instar larvae [118]. Other natural enemies, such as *M. basicornis*, *Campyloneuropsis infumatus*, *Engytatus varians*, and *Dicyphus* spp. put significant pressure on *Tuta absoluta* eggs and young larvae as well [119]. Direct biological control by predators, such as the ladybird beetle *Chrysoperla carnea*, or the predatory mites *Amblyseius cucumeris* and *A. swirskii*, feed on *Tuta absoluta* eggs and larvae directly [68,120]. Other natural enemies are additional predators, such as the ladybird beetles and lacewings, *Orius* and *Nabis*, and ground beetles, which predate a range of tomato pests and are strong natural biocontrol agents of pests, with a wide tropic range. They prey on whiteflies, unencrypted, and thrips, which are common in tomato crops [106].

Approximately 100 species of parasitoids, mainly egg endoparasitoids, larval endo- and ectoparasitoids, are known to parasitize *Tuta absoluta*. The recent works of Li et al. (2025) [121] demonstrated the possibility of augmentative biological control of the pest with some native Trichogramma parasitoids, especially *T. chilonis*. Such a discovery allows for the expansion of possibilities of IPM measures in China. However, the potential of the *Trichogramma* species, especially *Trichogramma cacoeciae*, originally from Africa, shows excellent and promising results [122]. The effectiveness of *T. cacoeciae* has been significantly explored in *Tunisia*, where this specific insect has been mass reared and released on the pests on the tomato plants in greenhouses and open fields. More than 5 million of these parasitoids are released thrice in one year to protect the pest damage, inhibit their reproduction, and reduce their population [71]. The complementary efforts of releasing the indigenous Trichogramma species have been used in the North African countries to manage *Tuta absoluta*. With the species capable of thriving on diverse hosts, such as parasitoids, it is essential to include them in pest regulation efforts, since there are different species causing damage globally.

Numerous larval parasitoids of *Tuta absoluta* have been reported mainly from the families *Bradconidae* and *Eulophidae* [123,124]. Notable endoparasitoids include *Pseudapanteles dignus*, *Dolichogenidea gelechiidivoris*, endoparasitoids attacking *Tuta absoluta* larvae, *Dineulophus phthorimaeae*, and *Necremnus tutae*, among others, offering the most promising potential for the control of *Tuta absoluta*, integral components of an IPM system in South America and Europe [7,125]. Local populations of the native *B. nigricans* as the ectoparasitoid of *Tuta absoluta* have been proposed as biocontrol agents in Africa, with some successful experimental trials having been performed in Tunisia, Kenya, and Sudan [123]. *D. gelechiidivoris* was introduced from Peru into sub-Saharan Africa as an endoparasitoid for *Tuta absoluta* through classical biological control [126]. In Spain and Algeria, it was accidentally introduced. *D. gelechiidivoris* is both a greenhouse and open-field parasitoid [92]. Recent findings have shown that non-reproductive parasitoid behaviors, such as host feeding and host interactions, dramatically influence host populations more than reproductive activities such as oviposition [127]. These novel insights demonstrate the necessity of clarifying parasitoid–host interactions and the importance of parasitoid behaviors on population ecology while developing biological control strategies.

Wang et al. (2024) [128] demonstrated the advantages of thelytokous lines of *N. formosa*, which exhibited higher parasitism rates, shorter developmental times, and increased fecundity compared to arrhenotokous lines. The present findings suggest that thelytokous lines of *N. formosa* may be more effective in reducing *Tuta absoluta* populations under field conditions, as they eliminate the reproductive costs associated with unproductive males. This trait enhances their efficiency in searching for and parasitizing *Tuta absoluta* larvae. The study also described the ovipositor insertion process, which was divided into stabbing, stirring, and oviposition phases. *Nissolia tutae* selectively feeds on first and second instar hosts, while ovipositing in third instars, with both feeding duration and oviposition time increasing with host larval age. These findings provide valuable insights into the host selection behavior of *N. tutae* and its role in improving biological control strategies against *Tuta absoluta*.

Entomopathogenic microorganisms, such as *Phthorimaea operculella* granulovirus (PhopGV), have also shown promise in *Tuta absoluta* management, with significant improvements in pest control when combined with *Bacillus thuringiensis* (Bt) and the conservation of Miridae predatory insects [83]. However, the combination of Bt and chemical insecticides is generally discouraged [129]. *Bacillus thuringiensis* var. *kurstaki* has been successfully used to control *Tuta absoluta* larvae in both open-field and greenhouse conditions [130]. Additionally, entomopathogenic nematodes, including *Steinernema carpocapsae* (Weiser), *S. feltiae*, and *Heterorhabditis* spp., have been shown to effectively infect larvae and reduce pest populations through soil and leaf applications [86]. *Metarhizium anisopliae* (Metsch.) has been reported to be effective against *Tuta absoluta* pupae [84], and both *B. bassiana* (Bals.) and *M. anisopliae* have been demonstrated to control a wide range of pests [131], as summarized in Table 1. These microbial agents present viable alternatives within integrated pest management strategies for *Tuta absoluta*. The comprehensive assessment of these methods is further illustrated in Table 1 and Figure 2.

### Enhancing T. absoluta Management with Sex Pheromones and Hormonal Control Strategies

Semiochemicals, specifically sex pheromones, are a benign approach to behaviorally regulated pest control because they comprise noxious signaling of conspecifics that does not result in continuous residues in harvested commodities or poisonous effects on the broader ecosystem. For *Tuta absoluta*, the important sex pheromone element is *3E*, *8Z*, *11Z*-tetradecatrienyl acetate; the synergistic minor component is tetradecadien-1-yl acetate. When used in relevant pheromone traps for monitoring, mass trapping, or mating disruption, pheromones are crucial elements in conjunction with further compatible technologies, such as biological variance and specific trapping, to further develop IPM and durability via organic protection over broad-spectrum chemistry [135]. South America, Europe, Asia, and North Africa have successfully managed leaf miners in shielded and unrestricted fields by creating carbon dioxide strategies. Many of these strategies could be transferable to Nepal. Pyriproxyfen, a juvenile hormone mimic that induces early insect death, has been effective in implementing varying geographical strategies [136]. Furthermore, by mixing sex-pheromone lures with specific insecticides, *Tuta absoluta* in tomato products has been hypothesized to be better controlled. Tomato systems (e.g., in Egypt) were indeed demonstrated to aid in the tautomerization of cells, implicating similar pheromones and technology-based completions as components of a larger, long-term strategy for organic development [137].

## 7. Unlocking the Power of Botanical Pesticides for *T. absoluta*

Multiple plant-derived compounds have shown potential as botanical insecticides targeting different developmental stages of *Tuta absoluta*, hence offering promising alternatives for its management. Multiple plant extracts, including chinaberry *Melia azedarach*, *Geranium nepalense*, onion *Allium cepa*, garlic *Allium sativum*, and jojoba *Simmondsia chinensis* seed extract, have recorded considerable larvicidal activity against *Tuta absoluta* larvae. The jojoba seed extract was effective against second-instar *Tuta absoluta* larvae [138]. Table 2 summarizes the botanical insecticides tested against different stages of *T. absoluta* and the larval instars’ levels of toxicity. Chinaberry extract’s efficacy level was the highest among the screened botanicals, followed by that of geranium, onion, and garlic, consequently [138]. Therefore, it is evident that the screened plant extracts can be explored as environmentally friendly technological options or in an integrated novel pest management approach.

Azadirachtin, a tetranortriterpenoid alkaloid derived from the neem tree, *Azadirachta indica*, has been widely explored for its multistage mechanisms of action in *Tuta absoluta*. Azadirachtin acts as an antifeedant, repellent, and inducer of sterility, which disrupts physiological processes that include oviposition, molting, and feeding, causing growth imbalances and density dependence in pests. The practical efficacy of azadirachtin is more notable in the first instars and becomes negligible from the third instar onward, into the last larval development stage [139]. This highlights the value of optimal timing for azadirachtin use in the pest’s life cycle.

**Table 2:** The botanical insecticides used against *Tuta absoluta*, together with information on plants and active compounds, and on the efficacy in pest control. It demonstrates the applications of these botanical insecticides as an alternate source in pest control.

Botanical	Insect Stage	References
*Azadirachta indica* A. Juss	Egg and Larva	Kona et al. (2014) [140]
*Allium sativum* L.	Second instar larva	Ghanim and Abdel Ghani (2014) [141]
*Eucalyptus globulus* Labill	Larva	Sanda et al. (2018) [142]
*Jatropa curcas* L.	Larva	Moreno et al. (2012) [118]
*Piper amalago* var. *medium*	Larva and pupa	(Brito et al., 2015) [143]
*Simmondsia chinensis*	Second instar larva	(Abdel-Baky and Al-Soqeer 2017) [138]
*Melia azedarach* L.	Second instar larva	(Ghanim and Abdel Ghani 2014) [141]
*Acmella oleracea* (L.) R.K. Jansen	All	(Moreno et al., 2012) [118]
*Allium cepa* L.	Second instar larva	(Ghanim and Abdel Ghani 2014) [141]

## 8. Evaluating the Effectiveness of Chemical Pesticides in Controlling *T. absoluta* Infestations

The efficacy of spinosad, emamectin benzoate, triflumuron, and diafenthiuron has been proven in managing *Tuta absoluta*. However, Ullah et al. 22 found that spinosad is highly toxic and causes intergenerational sublethal effects to the parental and next generations, and the pest may develop resistance from its indiscriminate and protracted use. For instance, spinosad, chlorantraniliprole, and novaluron are the chemicals recommended in Nepal for managing *Tuta absoluta*. These chemicals have their efficacy compromised by the broad host range of the pest, rapid population multiplication, and the development of resistance [144].

A study by Bastola et al. (2020) [145] in Palpa, Nepal, comparing the efficacy of spinosad, chlorantraniliprole, emamectin benzoate, and triflumuron showed the superiority of spinosad in minimizing leaf damage caused by *Tuta absoluta*, with no significant differences among the other three chemicals. The least effective chemical in reducing fruit damage in the four chemicals was chlorantraniliprole. The three chemicals, spinosad, emamectin benzoate, and chlorantraniliprole, showed some level of efficacy in reducing larval populations in the leaves and on fruits. A study by Simkhada et al. (2018) [146] from Kavresthali, Kathmandu, showed that chlorantraniliprole was more effective in reducing the number of leaf mines. Plant extracts can easily complement the action of these chemicals [147].

The frequent usage of chemical insecticides causes resistance development, thus resistance remains a major issue to the long-term success of sustainable pest management and crop production. Bastola et al. (2020) [145] reported indoxacarb’s low efficacy against the *Tuta absoluta* shock; populations exposed to indoxacarb had higher levels of resistance than those treated with chlorantraniliprole, emamectin benzoate, and spinosad. Therefore, reliance on chemical pesticides should not be a viable strategy. Thus, in order to avoid the development of resistance and guarantee the long-term successful *Tuta absoluta* control, rotation of chemicals can be performed frequently, and alternative pest management approaches can be included.

Resistance to commonly used insecticides against *Tuta absoluta* has been observed in various parts of the globe [148]. Pyrethroids, cartap, and organophosphates resistance have been reported from South America [149]. In Europe, there is extensive pyrethroid resistance and moderate indoxacarb, spinosyns, and spinosad resistance [150]. Diamides have been subjected to resistance in both Brazil and Europe, and a subsequent study revealed an altered target site insensitivity to chlorantraniliprole and spinosad occurring recently [116]. In addition, nano-encapsulated dsRNA targeting overexpressed P450 genes enhanced the susceptibility of the resistant *Tuta absoluta*, suggesting the genes’ involvement in resistance to tetraniliprole and cyantraniliprole. The rapid acquisition of insecticide resistance has urged the development of pest management into a significant challenge that requires early detection and mitigation. There is a possibility of preventing future resistance escalation through -based approaches by identifying high-risk susceptibility regions and spatial mapping to develop strategies for immediate intervention [151].

As the biocontrol mentioned above indicates, insecticides exhibit sublethal properties or acute toxicity detrimental to beneficial arthropods [152]. This is detrimental to IPM, limiting its effectiveness. An example in this review is the biocontrol mortality of *B. nigricans* due to a reduced concentration of chlorantraniliprole residual at over 35 °C [80]. Nevertheless, laboratory findings showed that there was no present harm to generalist predators and that chlorantraniliprole had minimal effects on the survival and reproduction of the predators [153]. The use of spinosad in Brazil drives *Tuta absoluta* to the verge of lethality, which causes a high death rate of the predatory avenger. In cases where adequate rainfall fails to take place, the insecticide is still present and active a month after the application, a significant effect on IPM [154]. Additionally, it is evident from the review that the egg numbers of flower bugs are reduced while the movement of earwigs and stink bugs is increased. Botanical insecticides such as azadirachtin have very little effect on killing natural enemies and are ecologically friendly.

## 9. Exploring Cutting-Edge Biotechnological Innovations for the Comprehensive Control of *Tuta absoluta*

Transition of conventional chemical-based pest management practices to sustainable, nonchemical methods: Agricultural practices should shift from conventional pest control, characterized by chemical sprays, to a greener, pollution-free environment [155]. In part due to biotechnological advances, which involve new tools and techniques from molecular biology and genetic engineering, the methods used to control pests continue to evolve [156,157]. The sterile insect technique, molecular breeding, RNA interference, CRISPR/Cas9-mediated genome editing, and nanotechnology are some of the innovative methods that have been analyzed. Nanotechnology, CRISPR/Cas9, RNAi, and molecular breeding are examples of innovative approaches with potential applications in controlling *Tuta absoluta* that serve as suitable alternatives to traditional methods

### 9.1. Advancing Tuta absoluta Control with Sterile Insect Technique

The Sterile Insect Technique (SIT) is a widely recognized pest control method that involves the mass release of sterile male insects to mate with wild females, resulting in infertile eggs. This method has been particularly effective in managing *Tuta absoluta*, owing in part to the species’ genetic homogeneity and non-parthenocarpic reproduction. However, it is important to note that the genetic homogeneity of *Tuta absoluta* is not absolute, and variations in genetic diversity can still occur in wild populations. As such, while SIT’s success is attributed to the species’ general genetic uniformity, other factors influencing its effectiveness must also be considered [6,158].

SIT works by disrupting the reproductive cycle of *Tuta absoluta* through the release of sterile males, which leads to a decrease in population as the eggs produced are infertile. This method has shown significant potential for pest control, as it results in negative population growth, thereby suppressing pest numbers. Furthermore, the concept of inherited sterility extends the utility of SIT, as it induces sterility in subsequent generations, contributing to the long-term suppression of *Tuta absoluta*. Nonetheless, it is essential to clarify that SIT’s success is influenced by various factors, including irradiation doses, timing, and environmental conditions, beyond just the sterile male release [159].

The success of SIT hinges on achieving optimal sterilization levels without compromising the quality of the irradiated males. In a study conducted with *Tuta absoluta* populations from Buenos Aires, Argentina, an irradiation dose of 200 Gy X-rays was found to be effective. This dose produced sterile females and partially fertile males. When released in a 15:1 male-to-female ratio, the irradiated males contributed to a significant reduction in population compared to control cages with untreated moths. However, the irradiation dose must be tailored to the specific genetic characteristics of the population being targeted, and further studies are needed to refine the balance between sterility induction and male quality [160].

Combining SIT with natural enemies, such as the mirid predator *T. cucurbitaceus*, could offer a complementary approach to enhance pest control. Zhou et al. (2024) demonstrated that a 300 Gy gamma radiation dose could successfully sterilize female *Tuta absoluta*, which would aid in the suppression of populations in the field when combined with natural predators [161]. This combined approach would offer a more sustainable solution by improving the long-term effectiveness of SIT, ensuring that pest populations are managed efficiently over time. Additionally, unlike chemical pesticides, SIT presents a resistance-free pest control strategy since insects cannot develop resistance to sterility. Furthermore, SIT’s lack of non-target effects makes it an environmentally sustainable option for pest management [33,162].

**Figure 3:**
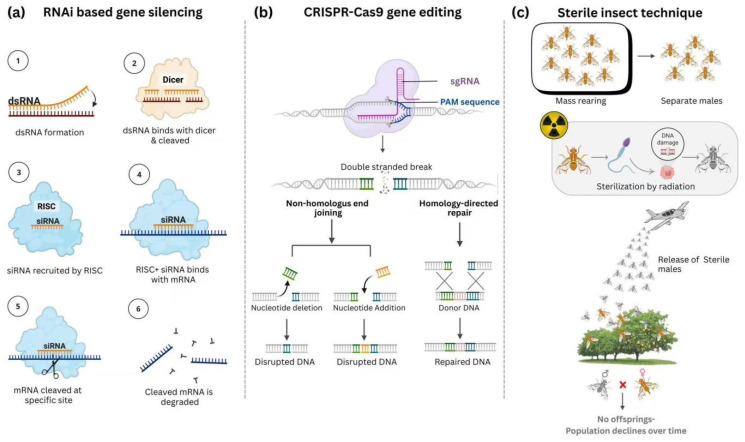
A comprehensive overview of three advanced genetic and biological techniques used for pest control. Figure (**a**) illustrates the process of RNAi-based gene silencing, where double-stranded RNA (dsRNA) is cleaved by an enzyme called Dicer into small interfering RNA (siRNA). This siRNA then binds with the RISC complex and targets a specific messenger RNA (mRNA), leading to its cleavage and degradation, effectively silencing the gene. Figure (**b**) depicts CRISPR-Cas9 gene editing, a powerful tool that uses a single guide RNA (sgRNA) to direct the Cas9 protein to a specific DNA sequence, creating a double-stranded break. The cell’s natural repair mechanisms, either non-homologous end joining (NHEJ) or homology-directed repair (HDR), are then used to either disrupt or precisely edit the gene. Figure (**c**) explains the Sterile Insect Technique (SIT), a form of pest control that involves mass-rearing insects, separating the males, and sterilizing them with radiation to induce DNA damage. These sterile males are then released into the wild, where they mate with wild females, but since the mating is infertile, no offspring are produced, leading to a decline in the pest population over time.

Despite its demonstrated potential, the scalability and cost-effectiveness of SIT remain critical concerns. Large-scale implementation of SIT requires careful evaluation of the economic feasibility of mass-rearing, sterilizing, and releasing insects. Furthermore, a comparison of SIT with other pest control methods, such as biological control or chemical treatments, is needed to assess its relative efficacy, cost, and scalability. While SIT shows promise as a tool for *Tuta absoluta* control, its limitations and potential for integration with other pest management strategies should be further explored. Future research should focus on optimizing irradiation protocols, refining release strategies, and integrating SIT with other pest control methods to enhance its effectiveness and scalability. Additionally, long-term studies are needed to assess the ecological impacts and potential unintended consequences of SIT, particularly when combined with other strategies [161]. In conclusion, while SIT remains a promising method for controlling *Tuta absoluta*, further research is necessary to optimize its application, assess its cost-effectiveness, and determine its long-term sustainability. A thorough, evidence-based comparison of SIT with alternative pest control strategies, along with a detailed research agenda focused on its scalability and integration with other methods, will be crucial for maximizing its impact in integrated pest management systems, as shown in Figure 3c.

### 9.2. RNAi-Mediated Gene Silencing for Targeted Control of Tuta absoluta

Double-stranded RNA (dsRNA)-mediated RNA interference (RNAi) has emerged as a promising insect pest control strategy, including for *Tuta absoluta*. RNAi works by silencing essential genes in *Tuta absoluta* larvae, leading to significant growth defects and increased mortality. However, while RNAi presents a viable approach, its practical application for pest management must be evaluated with a critical perspective, particularly concerning its scalability, environmental impact, and integration into broader pest management frameworks [163]. Plant-based induced transient gene silencing (*PITGS*) has been identified as a valuable means to enhance RNAi efficacy. By expressing dsRNA in crops, such as tomatoes, *PITGS* reduces pest infestation since the dsRNA is directly transferred to the pests via feeding. Studies have shown that dsRNA can be successfully delivered to tomato leaflets through *PITGS* or by direct uptake by *Tuta absoluta* larvae, demonstrating the potential of this method for pest suppression. Nonetheless, a more detailed evaluation of *PITGS’s* effectiveness in diverse field conditions and pest populations is necessary to assess its long-term viability and adaptability to various agricultural systems [164].

The potential for RNA-based biopesticides in integrated pest management (IPM) frameworks has been explored through studies on *Tuta absoluta*. Camargo et al. (2015) [165] demonstrated that target genes in *Tuta absoluta* are amenable to silencing, which further supports the use of RNAi as a biopesticide approach. RNA-based biopesticides offer distinct advantages over conventional chemical insecticides, including environmental safety, species specificity, and minimal non-target toxicity. For example, silencing the caspase-1 gene in *Tuta absoluta* using RNAi resulted in reduced larval body mass, inhibited growth, and disrupted development, underscoring RNAi’s potential as a non-chemical alternative for pest control. However, despite these promising findings, the scalability, cost-effectiveness, and field application of RNA-based biopesticides still require comprehensive analysis [166]. Furthermore, integrating RNAi with biological control agents such as *Nesidiocoris tenuis*, a natural predator of *Tuta absoluta*, could enhance the effectiveness of pest management strategies. The compatibility of RNAi with biological control methods provides an integrated approach that reduces reliance on synthetic chemicals while promoting sustainable pest control practices. This integration holds great promise for achieving long-term pest suppression, but a more critical review of how these approaches work together, and under what conditions they may be most effective, is essential.

Recent research by Yang et al. (2024) has identified 3-phosphoinositide-dependent protein kinase-1 (PDK1) as a crucial gene for *Tuta absoluta* development [167]. This gene plays an essential role in the larval-to-pupa transition, and its silencing leads to significant reductions in fecundity, fertility disruption, and morphological abnormalities in adults. The involvement of PDK1 in insect growth and reproduction makes it a promising target for new pesticidal compounds. Blocking PDK1 activity could dramatically suppress *T. absoluta* populations, offering a targeted strategy for pest management. However, further studies are necessary to evaluate the feasibility of targeting PDK1 in field conditions and to investigate potential off-target effects that could influence non-pest species in the ecosystem. In addition to PDK1, Yang et al. (2024) [167] also explored the role of the vitellogenin gene (TaVg) in *T. absoluta* reproduction. Silencing TaVg using RNAi resulted in females that lacked ovaries and were incapable of producing offspring, further demonstrating the potential of RNAi for disrupting essential reproductive pathways in *Tuta absoluta*. This discovery opens new avenues for genetic pest control strategies. However, as with PDK1, the long-term efficacy and ecological consequences of targeting TaVg in *Tuta absoluta* need to be rigorously tested in diverse field environments to ensure that the approach is both effective and sustainable.

Overall, RNAi-mediated gene silencing holds substantial promise for pest control due to its specificity, targetability, and environmental compatibility. However, there are critical gaps in understanding the scalability, cost-effectiveness, and ecological impacts of RNAi in pest management. Integrating RNAi with other control strategies, such as biological control and chemical alternatives, could provide a more holistic approach to pest management, but further research is needed to address the practical limitations and challenges of field application. A detailed, evidence-based assessment of RNAi’s long-term effectiveness, its integration into existing pest management practices, and its compatibility with other control methods is necessary to fully realize its potential as part of integrated pest management (IPM) frameworks, as illustrated in Figure 3a.

### 9.3. CRISPR/Cas9-Based Genome Editing

CRISPR/Cas9-mediated genome editing has rapidly emerged as a transformative biotech nology tool with vast potential applications in insect pest management. This system offers substantial advantages in functional genomics, particularly for species like *Tuta absoluta*, where such studies have previously been hindered. The ability to generate targeted mutations in *Tuta absoluta* provides an invaluable opportunity to study the functions of specific genes and their associated phenotypic outcomes, such as alterations in behavior, development, and reproduction. While the CRISPR/Cas9 system shows promise, its practical application in pest management requires further investigation into its scalability, efficiency, and ecological implications in the field.

CRISPR/Cas9-induced mutagenesis in *Tuta absoluta* has successfully generated phenotypic diversity, as seen with the modification of eye color patterns in larvae, demonstrating its potential for creating genetically modified strains of pests. This system enables rapid screening of genes involved in sex determination, fitness, and mortality, thereby offering new avenues for targeted pest control. However, while these findings are encouraging, it is essential to critically assess the long-term stability of CRISPR-induced traits and whether they will consistently produce the desired effects across diverse environments and generations of *Tuta absoluta*.

Ji et al. (2022) [168] demonstrated the successful application of the CRISPR/Cas9 system in *Tuta absoluta* by programming Tacinnabar gene mutagenesis, resulting in a red and mosaic eye color phenotype. This study represents the first successful application of gene editing in *Tuta absoluta*, validating the concept that CRISPR/Cas9 can be used effectively in this invasive pest species. While this breakthrough offers substantial promise, it is crucial to note that the practical applications of CRISPR/Cas9 in pest management require further refinement. For instance, while eye color mutations are a useful proof of concept, more research is needed to identify and edit genes that directly impact pest fitness, reproduction, and resistance to control measures.

The potential of CRISPR/Cas9 extends beyond single-gene mutations to more complex pest control strategies. For example, this technology could be used to create heritable mutations in *Tuta absoluta* that reduce its fitness, thereby diminishing its impact on crops such as tomatoes [169]. This approach is not limited to *Tuta absoluta*; similar applications have been successfully employed in other pest species like the fall armyworm and oriental fruit fly [170]. However, the long-term ecological consequences of releasing genetically modified pests into the environment must be carefully considered. Issues such as gene flow to non-target populations, unintended ecological impacts, and the development of resistance to CRISPR-induced modifications need to be addressed through rigorous field studies and monitoring.

Additionally, CRISPR/Cas9 holds significant promise for the development of advanced pest management techniques, including precision Sterile Insect Technique (SIT), gene drives, and CRISPR-engineered genetic sexing strains. These innovations could potentially enhance the effectiveness of *Tuta absoluta* control strategies by reducing population growth and reproductive success, leading to more sustainable pest suppression over time [171]. However, while the technology offers high precision in genetic modification, its scalability, cost-effectiveness, and ethical considerations surrounding the release of genetically modified organisms into the environment remain key challenges that require thorough investigation.

In conclusion, while CRISPR/Cas9 technology presents revolutionary potential for pest control, its practical application in integrated pest management (IPM) strategies for *Tuta absoluta* is still in the early stages. Critical gaps remain in understanding the long-term effectiveness, environmental risks, and ethical implications of deploying genetically modified pests in the field. Future research should focus on optimizing CRISPR/Cas9-based approaches for pest control, assessing their integration with other management strategies, and addressing potential ecological and regulatory challenges. As illustrated in Figure 3b, CRISPR/Cas9 could indeed become a pivotal tool in sustainable agricultural pest management, but its success will depend on a balanced and evidence-based approach to development and implementation.

### 9.4. Nano-Bioinsecticides for Tuta absoluta Control

The use of nanotechnology in the development of the nano-bioinsecticide to combat *Tuta absoluta* can thus be potentially applied in sustainable agriculture. Nano biopesticides have more significant opportunities, for example, more effective protection, high stability, re-targeted release, improved stress resistance, and environmental half-life [157]. By mixing bioactive substances such as Bacillus thuringiensis toxins, azadirachtin, pyrethrin, essential oils, and other substances that have already shown their effectiveness against a critical pest *Tuta absoluta*, with safe and non-toxic nanomaterials, it is possible to obtain more effective nano-bioinsecticides with strong protective action for tomatoes. For example, the cru-nano-formulation of citrus peel EOs has significant activity against *Tuta absoluta*.

Several studies have demonstrated that nano-based botanical insecticides, such as nanoemulsions, nanoparticles, and nanocapsule formulations, show enhanced insecticidal efficacy compared to traditional formulations without nanomaterials. Although Bt-based bioinsecticides have demonstrated activity on *Tuta absoluta*, the use of nano-formulation of *Bt* and/or encapsulating its crystals will be a new perspective in enhancing the efficacy and delivery systems of *Bt* crystal toxins, where field persistence and ultimately bio-efficacy will be improved by the nano-formulations. Several researchers have shown that cross-use of nanotechnology and genetic engineering has generated mesoporous silica nanoparticles and the cry1ab gene in transiently infected plants with the *Tuta absoluta* strain resistance plant [172].

Furthermore, with improved stability and delivery of the nano-carrier mediated system, dsRNA-based biopesticides for pest control have more bioactivity; hence, it is recommended for improved bioactivity [163]. However, the commercialization and application of nano-bio preparations would enhance *Tuta absoluta* control and ensure that innovative, sustainable agricultural management would be achieved. Therefore, nanotechnology in pest control is creating possible solutions to improve efficacy and stability, as well as the environmental safety of pest control approaches to be realized.

## 10. Future Perspectives

The persistent difficulty in controlling *Tuta absoluta* underscores the need for immediate, sustainable, high-tech, and high-precision pest control methods. With the increasing global prevalence and resistance of *Tuta absoluta* to chemical insecticides, combining novel biotechnologies with classical pest control methods will define the future of these controls. Some of the most promising technologies for future use include intensive RNA interference methods. Lethal RNAi-mediated biopesticides induce the destruction of unwanted pests while minimizing the exposure of non-target species to these genetic elements. More lethal RNAi targeting *Tuta absoluta* should be developed to reduce the dependence on chemical biocides. Additionally, CRISPR-Cas 9 technology also holds promise in controlling pests. With this biotechnology, genetically modified strains of *Tuta absoluta* could be developed with reduced fitness or sterility to achieve pest population control through the Sterile Insect Technique. Since these precise tools are used to destroy only the vital biological systems of the target organisms, they guarantee thorough work and reduce side effects. Moreover, nanotechnology shows promise in controlling *Tuta absoluta* through the development of nano-bioinsecticides. Nanotech-based biopesticides, including nanoformulations of *Bacillus thuringiensis* and plant extracts, offer intense formulations that stabilize and enhance the controlled discharge of biocides while increasing the life span of nano-biopesticides in the field. Biotechnological advances promise excellent potential for integrated pest management. The best outcomes will be derived from integrating genetic engineering, the use of genetic RNAi genes, bacterial microorganisms, and nanotechnology to promote pest-specificity with minimal unintended consequences or ecological side effects. Therefore, this prediction highlights how continued implementation of research, funding, and regulatory cooperation will be essential to enable the effective integration of these technologies, as well as translate their potential for real-world use on a large scale. These will then be designed for solution applications to assist farmers in adopting eco-friendly pest control methods; this will foster a novel, environmentally friendly era of pest control.

*Tuta absoluta* control continues to be a significant dilemma for global agriculture. However, the application of biotechnological techniques presents possibilities for breakthroughs. Particularly, RNAi, CRISPR/Cas9, and nanotechnology stand out as targeted and sustainable methods of control that reduce the reliance on chemical pesticides. When integrated into IPM protocols as part of the same tool, they could revamp the management of *Tuta absoluta* by enhancing the benefits and minimizing negative externalities. Research, development, and collaboration between parties involved within academia, industry, and regulatory agencies will be essential for realizing the full potential of this technology and translating it into practical solutions that benefit global agriculture in the long term.

## Figures and Tables

**Table 1:** Overview of the microbial agents evaluated against *Tuta absoluta*, including their type, mechanism of action, and efficacy in pest management. It focuses on diverse bacterial, fungal, and viral pathogens and their potency as biocontrol agents.

Microbial Agents	Insect Stage	References
*Beauveria bassiana*	Just hatched to the fourth instar	Giustolin et al. (2001) [132]
*Metarhizium anisopliae*	Larva and pupa	Sridhar et al. 2017 [133]
*Bacillus thuringiensis* var. *kurstaki*	Newly hatched, second, and third instar larvae	Shalaby et al. (2013) [113]
*Beauveria bassiana* Bals. Criv. *Metarhizium anisopliae* (Metchnikoff) Sorokın	Third instar larvae	Sabbour (2014) [134]
*Bacillus thuringiensis Berliner*	Second instar larvae	Halder et al. (2019) [114]

## Data Availability

No new data were created or analyzed in this study.

## Abbreviations

The following abbreviations are used in this manuscript:

SIT	Sterile Insect Technique
IPM	Integrated Pest Management
RNAi	RNA interference
CRISPR-Cas9	Clustered Regularly Interspaced Short Palindromic Repeats and CRISPR-associated protein 9.
EPNs	Entomopathogenic Nematodes
VOCs	Volatile Organic Compounds
NHEJ	Non-homologous End Joining
GAPDH	Glyceraldehyde-3-Phosphate Dehydrogenase
*N. tenuis*	*Nesidiocoris tenuis*
*S. carpocapsae*	*Steinernema carpocapsae*
